# Radiosurgery and Stereotactic Brain Radiotherapy with Systemic Therapy in Recurrent High-Grade Gliomas: Is It Feasible? Therapeutic Strategies in Recurrent High-Grade Gliomas

**DOI:** 10.3390/jpm12081336

**Published:** 2022-08-20

**Authors:** Fabiana Gregucci, Alessia Surgo, Roberta Carbonara, Letizia Laera, Maria Paola Ciliberti, Maria Annunziata Gentile, Morena Caliandro, Nicola Sasso, Ilaria Bonaparte, Vincenzo Fanelli, Romina Tortora, Eleonora Paulicelli, Giammarco Surico, Giuseppe Lombardi, Francesco Signorelli, Alba Fiorentino

**Affiliations:** 1 Department of Radiation Oncology, Miulli General Regional Hospital, 70021 Acquaviva delle Fonti (BA), Italy; 2 Department of Medical Oncology, Miulli General Regional Hospital, 70021 Acquaviva delle Fonti (BA), Italy; 3 Department of Radiology, Miulli General Regional Hospital, 70021 Acquaviva delle Fonti (BA), Italy; 4 Department of Neurosurgery, Miulli General Regional Hospital, 70021 Acquaviva delle Fonti (BA), Italy; 5 Centro Orientamento Oncologico, Miulli General Regional Hospital, 70021 Acquaviva delle Fonti (BA), Italy; 6 Department of Medical Oncology, Oncology 1, Veneto Institute of Oncology IOV-IRCCS, 35128 Padova, Italy; 7 Department of Basic Medical Sciences, Neurosciences and Sense Organs, Division of Neurosurgery, University “Aldo Moro”, 70124 Bari, Italy

**Keywords:** stereotactic radiotherapy, recurrence, glioma, chemotherapy, brain tumors

## Abstract

Purpose. For recurrent high-grade gliomas (HGG), no standard therapeutic approach has been reported; thus, surgery, chemotherapy, and re-irradiation (re-RT) may all be proposed. The aim of the study was to evaluate safety and efficacy of re-RT by radiosurgery or fractionated stereotactic radiotherapy (SRS/FSRT) in association to chemotherapy in patients with recurrent HGG. Material/Methods: All patients with histological diagnosis of HGG that suffered by recurrent disease diagnosed by magnetic resonance imaging (MRI), according to Response Assessment in Neuro-Oncology (RANO) criteria, after primary/adjuvant chemo-radiotherapy treatment and underwent to re-RT by SRS/FSRT were included in the analysis. Second-line chemotherapy was administered. Outcomes were evaluated by neurological examination and brain MRI performed 1 month after re-RT and then every 2–3 months. Results: From November 2019 to September 2021, 30 patients presenting recurrent HGG underwent re-RT. Median dose was 24 Gy (range 15–36 Gy), and median fractions was 5 (range 1–6). Twenty-one patients (70%) had RPA class ≤ IV. One patient had a histological diagnosis of anaplastic oligodendroglioma, 24 patients (80%) were affected by glioblastoma (GBM) including 3 cases of multifocal form, and 5 patients (17%) by anaplastic astrocytoma. Median time between primary/adjuvant RT and disease recurrence was 8 months. In six cases (20%) re-operation was performed, and in most cases (87%), a second line of systemic therapy was administrated. At a median follow-up time from recurrence of 13 months (range 6–56 months), 10 patients (33%) were alive: 2 patients with partial response disease, 7 patients with stable disease, and 1 patient with out-field progression disease. Of the 20 patients who died (67%), 15 (75%) died for progression disease and 5 (25%) for other causes (3 due to septic event, 1 due to thrombo-embolic event, and 1 due to car accident). Median OS and PFS after recurrence were 12.1 and 11.2 months. Six-month and one-year OS were, respectively, 81% and 51%. No acute or late neurological side effects grade ≥ 2 and no case of radio-necrosis were reported. One patient experienced, after reintervention and during Regorafenib treatment (administered 40 days after surgery), dehiscence of the surgical wound. In three cases, grade 2 distal paresthesia was reported. Grade 3–4 hematologic toxicity occurred in seven cases. Three case of grade 5 toxicities during chemotherapy were reported: three septic events and one thrombo-embolic event. Conclusion. Re-RT with SRT/FSRT in association with second-line systemic therapy is a safe and feasible treatment for patients with HGG recurrence. Validation of these results by prospective studies is needed.

## 1. Introduction

The most common primary brain tumors in adults are the high-grade gliomas (HGG) [1]. The standard of care at diagnosis is maximal safe surgical resection followed by adjuvant radio-chemotherapy with 60 Gy in 30 fractions and Temozolomide (TMZ) [2]. Unfortunately, despite this multimodal approach, prognosis remains poor: median overall survival (OS) is 12–18 months, and progression-free survival (PFS) is 6.8 months [2]. Considering the high incidence rate of recurrence within the initial tumor bed or irradiated volume and patients’ death for local progression, an improvement of local control should be sought [3,4,5]. Median OS after recurrence reported in literature ranges between 5 and 24 months [6,7,8,9,10,11,12], and there is no standard of care for salvage treatment after recurrence, and solely empirical indications exist. However, in the real-world data, most of patients are considered only for symptomatic supportive care. Nevertheless, in patients with good performance status reoperation, re-RT, second/third lines of systemic therapy, or a combination can be considered as salvage approach [13].

Comparing the genomic and molecular profiles of primary HGG with recurrence ones, disparities and inconsistencies have been demonstrated between the two entities; thus, for this reason, target therapy did not show an improvement. Chemotherapy with Lomustine or Regorafenib with or without Bevacizumab could be considered an option in cases with good performance status [14,15,16], reporting an overall survival of 5–8 months.

Reoperation with gross total tumor removal is the preferred treatment in selected patients (younger, good performance status, time of recurrence and size) and may provide a survival advantage [6,14], showing the important role of local therapy. Furthermore, reoperation for recurrent HGG has been associated with higher complication rates (morbidity until 70% and mortality 11%), precluding further therapeutic approaches [15,16]. As a consequence, more attention is given to minimally invasive salvage techniques.

By virtue of improvement in technology and techniques for radiotherapy (RT), re-RT may be considered one of the treatment modalities for HGG recurrence [17]. Fractionated stereotactic radiotherapy (FSRT) and radiosurgery (SRS) deliver a high-ablative radiation dose in a single or few fractions, providing a radiobiological advantage for radioresistant and recurrent tumors compared to standard RT regimens [18,19]. The high dose is extremely conformed to the tumor, sparing surrounding healthy tissue with a reduction of radiation-related side effects.

SRS has been demonstrating its role in secondary brain tumors as a substitute of surgery; thus, the hypothesis in recurrent HGG is to associate SRS and chemotherapy to improve outcomes. In fact, despite several retrospective series demonstrating the efficacy of SRS/FSRT [4,16,17,18,19,20,21,22,23,24], there is still insufficient evidence in favor of its use in association with chemotherapy.

In our Advanced Radiation Therapy department, a prospective data collection on new technical approach was performed; therefore, based on the above background, the present study was conducted to evaluate the safety and feasibility of re-RT by SRS/FSRT in association with systemic therapy in patients with recurrent HGG.

## 2. Materials and Methods

Based on prospective collected data about SRS/SFRT (approved by Institutional Review Board), we evaluated all patients with histological diagnosis of HGG who, in the study period, suffered from recurrent disease diagnosed by magnetic resonance imaging (MRI), according to the Response Assessment in Neuro-Oncology (RANO) criteria [25] after primary/adjuvant chemo-radiotherapy treatment and underwent to re-RT by SRS/FSRT. At the time of recurrence, all cases were discussed at a multidisciplinary team (MDT) meeting, and a consensus decision was made according to international guidelines [13].

In the case of unresectable disease or patient refusal of reintervention, patients were evaluated by a radiation oncologist for re-RT. 

Inclusion criteria were as follows: more than 18 years of age; Karnofsky performance scale (KPS) more than 50; and adequate bone marrow and renal and liver function. SRS or FSRT were performed with variable doses and fractions according to previous RT, size, and number of lesions and organs at risk (OaRs) proximity. To perform SRS/FSRT, patients underwent a simulation procedure: they were immobilized in the supine position with dedicated thermoplastic masks—Solstice^TM^ SRS Immobilization System (CIVCO^®^ Radiotherapy) or QFix^®^, Avondale, PA–USA [26,27,28]—and a computed tomography (CT) was performed without contrast, including the whole brain, acquiring slices of 1 mm thickness, as recently published [27,28]. 

For the contouring, co-registration with MRI was mandatory. OaRs were contoured: brain (normal brain minus PTV), eyes, lens, optic chiasm, optic nerves, brainstem, and spinal cord. The gross tumor volume (GTV) was defined on contrast-enhanced T1-weighted sequence MRI and was assumed equal to the clinical target volume (CTV). The planning target volume (PTV) was obtained from the GTV plus an isotropic margin of 1 mm in all directions. Volumetric dose prescription (Dp) to PTV was adopted by normalizing 100% Dp to 98% of the volume, while large intra-target dose heterogeneity D2% (PTV) < 150% Dp was accepted. According to the linear-quadratic model, an evaluation of cumulative dose for OaRs was performed, ensuring an equivalent uniform dose in 2 Gy fraction (EQD2) sum inferior to 100–120 Gy [29]. For RT planning, 6X flattening filter free and volumetric modulated arc therapy (VMAT) plans were generated with two or more coplanar or non-coplanar partial arcs by TrueBeamTM (Varian Medical System). In cases of multiple lesions, HyperArc^TM^ approach [26] was used.

During RT, IGRT with cone-beam CT (CBCT) and real-time surface-guided RT using AlignRT^®^ was performed daily prior to and during the RT session [26,27,28]. Re-RT was administrated before starting the second-line systemic therapy or between its first and second cycle.

During the follow-up, clinical examination and brain MRI were performed 1 month after reRT and then every 2–3 months. At each visit, neurological status and the severity of complications were rated according to the National Cancer Institute Common Toxicity Criteria (NCI-CTC ver. 4). Adverse neurological events were considered consequences of treatment in the absence of progressive disease. The RANO response criteria were adopted to evaluate disease status [25]. 

In case of progression, patients received a third re-RT and/or a third line of systemic therapy if indicated or best supportive care.

The first endpoint of this study was safety (acute and late toxicity), and the secondary endpoints were OS and PFS.

Informed consent was obtained from all patients included in the study. 

### Statistical Analysis

The outcome variables were acute and late toxicity, PFS, and OS. The acute and late toxicity were considered as categorial variables, defined according to NCI-CTC scale; moreover, the presence/absence of radio-necrosis was recorded as dichotomous variable. The PFS was calculated from the date of second surgery or re-RT to the time of progression or last follow-up date. The OS was calculated from the date of second surgery or re-RT to death for disease progression or last follow-up date. The Kaplan–Meier method was used to evaluate PFS and OS. A log-rank test was used to compare the different subgroups in univariate analysis. Multivariate analysis was performed to determine the independent prognostic factors by using Cox regression model. A two-sided *p*-value equal to or less than 0.05 was considered statistically significant.

All the following prognostic factors were evaluated: sex, age (<54 *vs.* ≥54), KPS (<80 *vs.* ≥80), RPA (III + IV *vs.* V), surgical resection (complete vs. incomplete), histological diagnosis (glioblastoma *vs.* others), O6-methylguanin-DNA-methyltransferase (MGMT) promoter methylation status (methylated *vs.* not methylated), adjuvant RT dose (conventional *vs.* hypofractionated), biological equivalent dose (BED) of RT at recurrence (<40 Gy *vs.* >40 Gy), PTV dimension (≤14.5 cc >14.5 cc), more local treatments (surgery + reRT *vs.* re-RT alone), recurrence time (<8 months *vs.* ≥8 months), and progression time (≤11 months *vs.* >11 months).

## 3. Results

From November 2019 to September 2021, 30 patients with recurrent HGG were evaluated in our department. Table 1 reports clinical characteristics of the study population.

At diagnosis, median age was 54 years (range 36–76), and median KPS was 80% (range 50–90%). Twenty-one patients (70%) had RPA class ≤ IV. One patient had a histological diagnosis of anaplastic oligodendroglioma, twenty-four patients (80%) were affected by glioblastoma (GBM) including three cases of multifocal form, and five patients (17%) by anaplastic astrocytoma. In 40% of cases, MGMT promoter was unmethylated, and in 80% of cases, the IDH1/2 gene was wild-type. 

Regarding treatment, at diagnosis, 13 patients (43.3%) had subtotal resection, and 3 patients (10%) received lesion biopsy alone, while MRI-confirmed complete resection was obtained in 14 cases (46.7%). All patients before relapse underwent primary/adjuvant RT with concomitant temozolomide (TMZ), with different fractionation approaches: 21 (70%) received conventional fractionated RT [2], and 9 (30%) received hypofractionated RT [28]. 

**Figure 1:**
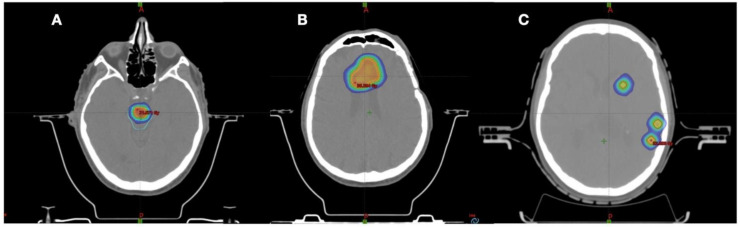
Examples of re-irradiation treatment plans. (**A**) Glioblastoma recurrence in brain stem treated with SFRT 18 Gy in three fractions, (**B**) glioblastoma recurrence treated with SFRT 30 Gy in five fractions, (**C**) multiple lesions for young patients with oligodendrogliomas treated with SFRT with HyperArc approach 18 Gy in three fractions.

All patients suffered by disease recurrence: the median time occurring between primary/adjuvant RT and disease recurrence was 8 months (range 2–27). At the time of recurrence, all patients underwent re-RT, receiving stereotactic radiotherapy with a median dose of 24 Gy (range 15–36 Gy) and median fractions of 5 (range 1–6). The median BED_10_ was 37.5 Gy (28–81.6 Gy). The median PTV was 14.5 cc (range 0.6–108.8 cc). In six cases (20%), a reintervention was performed. Most patients (87%) received a second line of systemic therapy, while four patients did not (one for not indication, one for refusal, and two for poor general clinical conditions). In Table 2 and Figure 1, treatment characteristics of the study population are summarized.

At a median follow-up time from recurrence of 13 months (range 6–56 months), 10 patients (33%) were alive: 2 patients with partial response disease, 7 patients with stable disease, and 1 patient with out-field progression disease. Of the 20 patients who died (67%), 15 (75%) died due to progression disease and 5 (25%) due to other causes (3 due to septic event, 1 due to thrombo-embolic event, and 1 due to car accident).

During follow-up, nine patients with out-field progression disease and good performance status underwent a third re-RT, with a median dose of 21 Gy (range 14–27 Gy) and median fractions of 3 (range 1–5).

**Figure 2:**
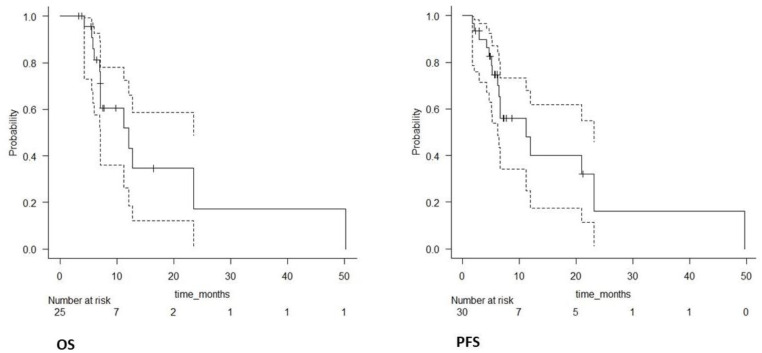
OS and PFS Kaplan–Meier survival curves of the entire population (solid lines); confidence intervals (dotted lines).

The median OS after recurrence was 12.1 months (95% CI 7.1–23.5); 6-month and 1-year OS were, respectively, 81% (95% CI 57–93%) and 51% (95% CI 26–72%). The median PFS after recurrence was 11.2 months (95% CI 6.2–23.1); 6-month and 1-year PFS were, respectively, 70% (95% CI 48–84%) and 32% (95% CI 13.12–52.8%). OS and PFS Kaplan–Meier survival curves are shown in Figure 2.

Regarding toxicity, no acute or late neurological side effects more severe than grade 2 were reported. No case of radio-necrosis was detected. In all cases, prophylactic steroid therapy was administrated. One patient, after reintervention and during Regorafenib treatment (administered 40 days after surgery), experienced a dehiscence of the surgical wound. In three cases, grade 2 distal paresthesia was reported. Grade 3–4 hematologic toxicity (neutropenia and thrombocytopenia) occurred in even cases. Three case of grade 5 toxicities during chemotherapy were reported: three septic events and one thrombo-embolic event. 

Neurocognitive assessment pre- and post-re-RT was available in a limited number of patients (10%) and therefore unreliable.

### Prognostic Factors for OS and PFS

Univariate and multivariate prognostic factors influencing OS are shown in Table 3. Four significant variables in univariate analysis (age, RPA, resection, and progression time) were entered into the multivariable model. As a result, age ≥ 54 years (HR: 7.98, 95% CI 0.76–83.0, *p* = 0.05) and incomplete resection (HR: 14.65, 95% CI 1.13–190.0, *p* = 0.04) were significant negative prognostic factors for survival, while progression time > 11 months (HR: 0.15, 95% CI 0.02–0.82, *p* = 0.02) was a significant positive prognostic factor for survival. None of the explored prognostic factors in univariate and multivariate analysis was significantly correlated with PFS.

## 4. Discussion

For the management of HGG recurrence, no standard salvage treatment has shown its superiority, and thus, treatment options including surgery, second-line chemotherapy, re-RT, and best supportive care should be proposed. Recently, Lombardi et al. in a phase II randomized trial that involved 119 recurrent GBM patients reported a median survival of 7.4 months for patients receiving Regorafenib compared to 5.6 months for patients receiving Lomustine as a second-line chemotherapy [16]. A retrospective study conducted by the neuro-oncology group of the Italian Association of Radiotherapy and Clinical Oncology showed a median OS of 9.7 months for 300 re-irradiated glioma patients (some patients with SRT) and, in particular, 8 months for GBM histology [17]. 

SRT, delivering a high-ablative dose, has the benefit of being a noninvasive procedure, and it could be an alternative to salvage surgery. However, considering the retrospective nature of the studies employing stereotactic re-RT for HGG recurrence, no robust data are present in the literature supporting SRT as the standard of care in recurrent HGG [4,20,21]. 

Previous studies showed a survival benefit of SRT compared to observation, reporting an increased OS between 2 to 8 months [18,30]. Kondziolka et al. [31] reported a median OS of 30 months from the time of initial diagnosis in 64 recurrent GBM patients re-irradiated with SRT, while other analyses [32,33,34] found a median OS of 8–12.5 months after salvage SRT for recurrent GBM. 

Generally, the interval between initial diagnosis and recurrence is considered as a prognostic factor for survival [35,36,37,38]; however, a precise interval is not highlighted. In the present analysis, the OS and PFS were longer if the time interval between initial diagnosis and recurrence was superior to 8 months. 

Another clinical prognostic factor in the literature is tumor volume. A PTV with a diameter superior to 40 mm was an independent negative prognostic factor for survival [7,39], as also reported in the pooled analysis of the EORTC Brain Tumor Group clinical trials [38]. In the Chapman study, which analyzed the re-irradiation in 116 HGG patients, the ROC analysis identified KPS ≤ 80%, age at re-irradiation ≥ 55 years, time to initial progression ≤ 12 months, and PTV volume > 6.4 cc for SRS and > 131 cc for non-SRS treatments as thresholds for reduction of outcome [40]. On the other hand, another study did not show correlation between tumor volume and survival after salvage SRT [16]. The present data similarly showed that a median value of 14.5 cc and prognostic analysis did not highlight any impact of PTV volume on OS (HR 1.09, 95% CI 0.33–3.62, *p* 0.88). 

Regarding radiation dose, no definitive data are reported [16,17]. In our analysis, the median re-RT dose was of 24 Gy (range 18–36 Gy) with median BED_10_ of 37.5 Gy (28–81.6 Gy). The prognostic analysis did not show any impact of BED_10_ on survival (BED_10_ > 40 Gy HR 0.42, 95%CI 0.08–1.99, *p* 0.27) (BED_10_ > 50 Gy HR 0.55, 95% CI 0.14–2.19, *p* 0.38). Navarria et al., considering the wide range of total doses and fractionations used for 300 re-irradiated patients, tried to identify a threshold of a biological effective dose on the tumor (BED10) impacting survival, and a BED10 threshold > 43 Gy proved to influence OS [17]. In a retrospective study including 19 patients [33], OS was longer after a total dose of 30 Gy versus <30 Gy (11.1 *vs.* 7.4 months, *p* = 0.051). However, a recent meta-analysis did not show any dose–response relationship with OS [39]. Regarding our study population, all 16 patients with progression of disease experienced an SRS/FSRT out-field recurrence; thus, total dose used was correlated neither to PFS nor to OS. 

Regarding the association of chemotherapy with SRT, the data are inconclusive and sparse. Conti et al. [41] evaluated the effect of TMZ together with salvage SRT in 23 recurrent GBM patients; in the analysis, 11 patients underwent SRT alone, and 12 patients underwent SRT with TMZ at 75 mg/m^2^/day for 21 days every 28 days. Median OS and PFS were significantly better in the combined arm with respect to SRT alone (12 vs. 7 months, *p* < 0.01; 7 vs. 4 months, *p* = 0.01, respectively). The same results were reported if SRT was combined with bevacizumab for recurrent GBM patients (median OS: 8.6 vs. 5.7 months) [42]. The latter results are confirmed also in the present analysis: 26 out 30 patients received chemotherapy, and our median OS was 12 months. A recent trial on re-irradiated patients with SRT reported no impact on OS and PFS in 33 patients who received sequential chemotherapy after SRT [34]. A recent meta-analysis included 50 studies and more than 2000 re-irradiated patients. The authors found no significant difference in terms of outcome if concurrent systemic therapy was added to re-irradiation [43]. It is important to underline that only 15 papers out of 50 reported data on chemotherapy and re-irradiation using Temozolomide, Lomustine, or Bevacizumab as a single agent and that few patients were treated by SRT. In the present analysis, 17 out 26 patients received Regorafenib as a second-line chemotherapy. The recent phase II trial by Navarria et al. on 90 re-irradiated patients [44] reported a median OS of 17 months (14 for GBM). However, only 53% of cases received chemotherapy, and only 17 out 90 received SRT, of which only 11 patients received chemotherapy (Lomustine, procarbazine, temozolomide).

Regarding salvage surgery, in the present analysis, six patients were reoperated. In several surgical trials, re-operation was associated with a median OS of 6 months, without significant benefit [14,44,45,46]. Park et al. [47] investigated preoperative risk factors for recurrent GBM patients, showing that tumor location near critical structures, poor KPS score, and high tumor volume (≥50 mL) correlated with poor survival after redone surgery. In the study of Yaprak et al., seven re-operated patients before salvage SRT had a significantly poorer OS after SRT compared to un-operated ones (*p* = 0.02) [34]. De Bonis et al. reported an increased survival when redone surgery was administered to recurrent GBM; however, the authors reported that patients with a KPS < 70 were significantly at risk of death (HR 2.8—*p* = 0.001) [48]. Post-surgical morbidity and mortality are important concerns, and poor survival of recurrent GBM patients necessitates careful preoperative risk assessment and good selection of patients. In the present analysis, patients undergoing reintervention had a median age of 50 years, median KPS of 90, median disease volume of 12 cc, and had a better survival compared to the whole study population, with a median OS of 21 months. In fact, the accuracy in eligible patients’ selection for salvage surgery is relevant. According to the literature [47,48,49,50], younger age and better performance status, size, and location of recurrence are relevant factors to take into account for reoperation. Moreover, it is important to consider the association with systemic therapy and possible impact of side effects. In our experience, one patient that received Regorafenib at 45 days after reintervention presented with surgical wound dehiscence, requiring interruption of treatments. 

A concern regarding re-RT is the risk of radio-necrosis. In a study investigating the role of salvage SRT in 84 patients, the authors did not observe any radiation necrosis [49]. In the present study, SRT was well-tolerated, and no grade III/IV treatment-related toxicity was noted throughout the follow-up period.

## 5. Conclusions

In patients with recurrent HGG, limited data support a standard of care [51]. The resent analysis indicates that re-RT with a high-ablative dose in few fractions may be offered in select patients with good performance in association with second-line systemic therapy, including Regorafenib. Re-surgery in young patients with good performance status and low burden recurrence could also improve the outcome. Despite the limitations of the present analysis (retrospective analysis without control group with a relatively small sample size for whom the extent of first resection and type of treatment at relapse were not homogeneous), the data of OS (12 months) by SRT and chemotherapy, including the new regorafenib, are encouraging compared to chemotherapy alone (5–7 months). Thus, further investigations with larger series are warranted for defining the role of SRS plus chemotherapy in recurrent HGG. The future analysis should be performed also for better definition of target volume, SRT dose and fractionation, timing of systemic therapy, and eventually association of re-RT with newer targeted therapies.

## Figures and Tables

**Table 1:** Patients and tumor characteristics.

Number of patients		30	
Gender			
	Male	19	(63%)
	Female	11	(37%)
Age			
	Median (range) (years)	54	(36–76)
	<60 (years)	22	(73%)
	60–65 (years)	5	(17%)
	>65 (years)	3	(10%)
KPS score			
	Median (range) (%)	80	(50–90)
	<60%	1	(3%)
	60–70%	11	(37%)
	>70%	18	(60%)
RPA class			
	Median (range)	IV	(III–V)
	III	6	(20%)
	IV	15	(50%)
	V	9	(30%)
Mass effect			
	Yes	16	(53%)
	No	14	(47%)
Multifocal tumor			
	Yes	3	(10%)
	No	27	(90%)
Tumor histology			
	Glioblastoma	24	(80%)
	Anaplastic Astrocytoma	5	(17%)
	Oligodendroglioma	1	(3%)
MGMT methylation			
	Methylated	10	(33%)
	Unmethylated	12	(40%)
	Not Available	8	(27%)
IDH Mutation			
	Mutated	0	(0%)
	Wild Type	24	(80%)
	Not Available	6	(20%)

**Table 2:** Treatment characteristics for all population.

**Number of patients**	30	
**Surgery at diagnosis**		
Complete	14	(47%)
Incomplete	13	(43%)
Unresectable (biopsy)	3	(10%)
**Median time between surgery and adjuvant therapy (range)**	8 weeks	2–18 weeks
**Primary/Adjuvant RT + TMZ**		
Hypofractionated	9	(30%)
Conventional fractionated	21	(70%)
**Median time between adjuvant therapy and recurrence (range)**	8 months	2–27 months
**Surgery at recurrence**		
Performed	6	(20%)
Not performed	24	(80%)
**reRT at recurrence**		
Performed	30	(100%)
**Systemic therapy at recurrence**		
Regorafenib	17	(57%)
Fotemustine	5	(16%)
Bevacizumab	2	(7%)
Metronomic Temozolomide	2	(7%)
None	4	(13%)

**Table 3:** Univariate and multivariate analysis of prognostic factor for overall survival.

Variable	Univariate			Multivariate		
	HR	95% CI	*p*	HR	95% CI	*p*
Sex	5.88	0.75–46.06	0.09	-	-	-
Age (≥54 years)	3.8	0.9–16.02	0.02	7.98	0.76–83.0	0.05
KPS (≤80%)	0.65	0.15–2.76	0.5	-	-	-
RPA (≥IV)	6.64	0.83–52.69	0.05	3.78	0.42–33.64	0.2
Adjuvant RT dose (hypofractionated)	3.66	0.7–19.01	0.12	-	-	-
Resection (incomplete)	5.32	1.1–27.48	0.04	14.65	1.13–190.0	0.04
Diagnosis (GB)	0.67	0.14–3.16	0.61	-	-	-
MGMT methylation (absent)	0.56	0.11–2.81	0.48	-	-	-
BED of RT dose at recurrence (>40 Gy)	0.42	0.08–1.99	0.27	-	-	-
PTV (>14.5 cc)	1.09	0.33–3.62	0.88	-	-	-
More local treatment	0.37	0.09–1.44	0.15	-	-	-
Recurrence time (≥8 months)	1.69	0.5–5.7	0.39	-	-	-
Progression time (>11 months)	0.12	0.02–0.64	0.01	0.15	0.02–0.82	0.02

## Data Availability

The data can be acquired from the corresponding author.
